# Plant antimicrobial peptides: structures, functions, and applications

**DOI:** 10.1186/s40529-021-00312-x

**Published:** 2021-04-29

**Authors:** Junpeng Li, Shuping Hu, Wei Jian, Chengjian Xie, Xingyong Yang

**Affiliations:** College of Life Science, Chongqing Normal University, Chongqing, 401331 China

**Keywords:** Thionins, Defensins, Hevein-like peptides, Knottins, Α-hairpinins, Lipid transfer proteins, Snakins, Cyclotides

## Abstract

Antimicrobial peptides (AMPs) are a class of short, usually positively charged polypeptides that exist in humans, animals, and plants. Considering the increasing number of drug-resistant pathogens, the antimicrobial activity of AMPs has attracted much attention. AMPs with broad-spectrum antimicrobial activity against many gram-positive bacteria, gram-negative bacteria, and fungi are an important defensive barrier against pathogens for many organisms. With continuing research, many other physiological functions of plant AMPs have been found in addition to their antimicrobial roles, such as regulating plant growth and development and treating many diseases with high efficacy. The potential applicability of plant AMPs in agricultural production, as food additives and disease treatments, has garnered much interest. This review focuses on the types of plant AMPs, their mechanisms of action, the parameters affecting the antimicrobial activities of AMPs, and their potential applications in agricultural production, the food industry, breeding industry, and medical field.

## Introduction

Throughout their life cycle in natural ecosystems, plants usually coexist in an environment rich in a wide variety of microorganisms and pests. During their evolution, to protect them from the damage of pathogens and pests, plants have developed sophisticated defense measures that enable them to effectively defend against deleterious organisms such as bacteria, fungi, nematodes, and insects (Iqbal et al. 2019; Yang et al. 2007). These defense measures against pathogens include physical barriers against their penetration and spread, such as waxy cuticle layers and trichomes, and chemical barriers to inhibit pathogen growth and development, such as complex cell recognition systems, intricate phytohormone networks, innumerable transcriptional pathways, diverse proteins, and secondary metabolites (Campos et al. 2018). Among plant defense molecules, antimicrobial peptides (AMPs) are one of the most common and prominent chemical barriers that plants have developed to resist biotic stresses (Kulaeva et al. 2020).

AMPs, which are part of the innate immune system inherent in almost all lifeforms, including microorganisms, arthropods, animals, and plants, contribute greatly to the host defense against pathogens (Zasloff 2002). In general, AMPs are small molecule polypeptides synthesized by ribosomes, in which the production of the mature polypeptides involves cleavage from larger protein precursors and further post-translational modifications. In addition, some AMPs can be synthesized through non-ribosomal peptide synthetases (Mukherjee et al. 2011; Tyagi et al. 2019). Many AMPs exhibit a broad-spectrum antibiotic activity against pathogenic bacteria (gram-negative and gram-positive), fungi, enveloped viruses, and parasites (Havenga et al. 2019; Marcocci et al. 2020; Zahedifard et al. 2020). In humans and other higher organisms, AMPs are an important part of the innate immunity in response to pathogenic bacterial infections, and they are the first line of defense for various organisms against infections of multiple pathogenic microorganisms (Tang et al. 2018; Zasloff, 2002). For single-cell organisms, AMPs may offer an advantage to compete with organisms with similar nutritional and ecological needs (Hegedüs and Marx, 2013). Although AMPs vary between species, they have similar sequence and structural characteristics, such as possessing shorter lengths (generally only 12–50 amino acids), positive charges, and hydrophobic and hydrophilic regions (Tam et al. 2015). AMPs can usually target multiple sites on the plasma membranes and intracellular components of pathogens, but they show low cytotoxicity to mammals such as humans and swine (Kohn et al. 2018; Lian et al. 2020; Taggar et al. 2021). AMPs can quickly kill multidrug-resistant pathogens at low concentrations, and microorganisms have a low tendency to develop resistance to them (Park et al. 2011). As potential drugs for the treatment of many infectious and fatal diseases, research on AMPs in the field of medicine has been intensifying (Ciociola et al. 2016; Melo et al. 2009).

**Fig. 1:**
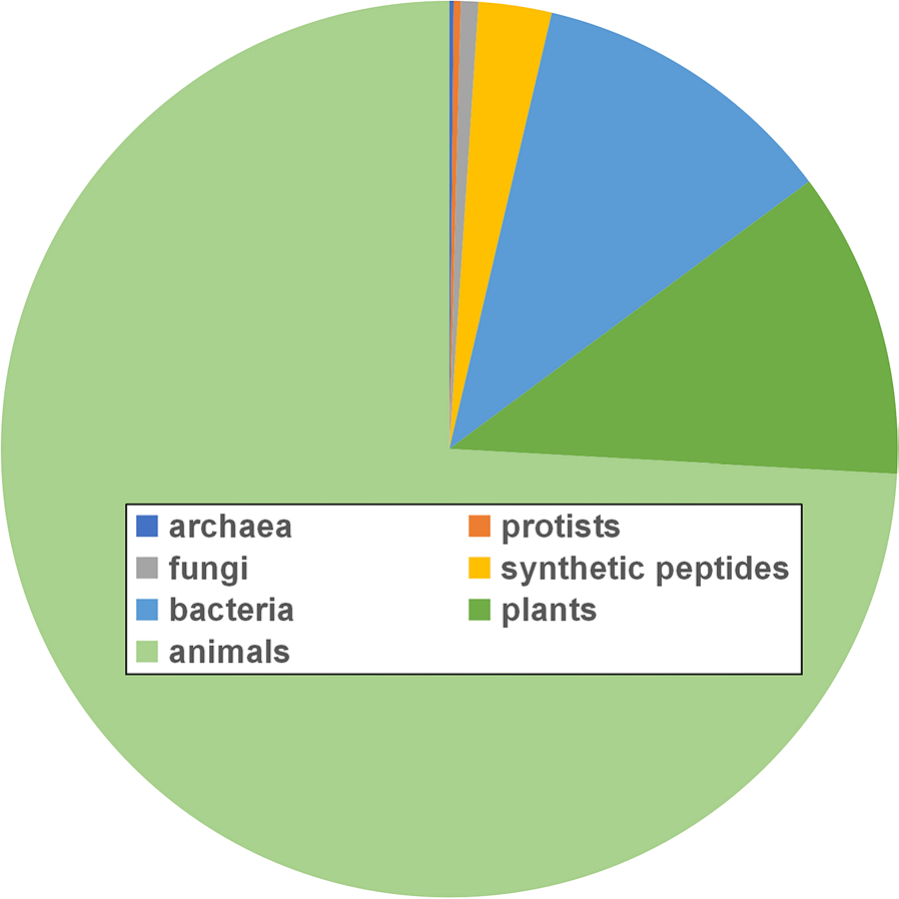
Sources of antimicrobial peptides from the antimicrobial peptide database (http://aps.unmc.edu/AP/)

AMPs have been mined from all areas of life. To date, more than 3000 AMPs have been isolated from microorganisms, insects, amphibians, plants, and mammals (Fig. 1). Plants are a promising source of AMPs, and these peptides have significant antimicrobial activity against both human and plant pathogens. It is generally believed that AMPs can be produced in different parts of plants (leaves, roots, seeds, flowers, and stems) (Tam et al. 2015; Wang et al. 2016). Since the first report of the plant AMP thionin from *Triticum aestivum* (Fernandez et al. 1972; Hughes et al. 2000), research on plant AMPs has been developing rapidly. Plant AMPs are considered not only to play a role in plant defenses against pathogens but also to significantly affect plant growth and development (Berrocal-Lobo et al. 2002; de Zélicourt et al. 2007; Fernandez et al. 1972). In addition, with the increasing resistance of various pathogenic bacteria to common antibiotics, there are some plant AMPs that continue to be beneficial in the treatment of various fatal human diseases. Therefore, the application of AMPs in the treatment of diseases in the medical field is receiving increasing attention.

## Classification of plant AMPs

The composition of plant AMPs is very complex. A single plant species can contain a variety of AMPs (Noonan et al. 2017). Most plant AMPs are positively charged at physiological pH with molecular weights ranging from 2–10 kDa. AMPs contain 4–12 cysteine residues forming disulfide bonds, which can make them exceptionally stable to chemical, thermal and enzymatic degradation by stabilizing their tertiary and quaternary structures (Faull et al. 2020; Khoo et al. 2011; Sohail et al. 2020; Tam et al. 2015). The smallest known AMP comprising seven amino acids (Lys-Val-Phe-Leu-Gly-Leu-Lys) was isolated from *Jatropha curcas* (Xiao et al. 2011). Plant AMPs have diverse functions, structures, and expression patterns, as well as specific targets, which make their classification more complex and difficult. Plant AMPs can be classified into cationic peptides and anionic peptides according to their charge (Hammami et al. 2009; Prabhu et al. 2014). However, the classifications of plant AMPs are usually based on their sequence similarity, presence of cysteine motifs, and tertiary structures (Hammami et al. 2009) (Table 1).

**Table 1:** Summary of some common plant antimicrobial peptides about their classification and Bioactivities

Peptide	Classification	Function	Potency	Refs.
Purothionins	thionin	Antibacterial	1–540^b^	Fernandez et al. 1972
Cp-thionin II	thionin	Antifungal	50^b^	Schmidt et al. 2019
CaThi	thionin	Antibacterial, Antifungal	10–40^f^	Taveira et al. 2014
alfAFP	Plant defensin	Antifungal	5^f^	Gao et al. 2000
Fa-AMP1	Plant defensin	Antibacterial, Antifungal	11^f^	Fujimura et al. 2003
Fa-AMP2	Plant defensin	Antibacterial, Antifungal	36^f^	Fujimura et al. 2003
*C. fistula* PI	Plant defensin	Trypsin inhibitor	2^e^	Wijaya et al. 2000
Rs-AFP1	Plant defensin	Antifungal	5-15^f^	Terras et al. 1995
Rs-AFP2	Plant defensin	Antifungal	2^f^	Terras et al. 1995
Rs-AFP3	Plant defensin	Antifungal	2^f^	Terras et al. 1995
Rs-AFP4	Plant defensin	Antifungal	5–11^f^	Terras et al. 1995
Vv-AMP1	Plant defensin	Antifungal	3.6–15^f^	de Beer and Vivier, 2008
ZmD32	Plant defensin	Antibacterial, Antifungal	0.5–4.0^e^	Kerenga et al. 2019
NaD1	Plant defensin	Antibacterial, Antifungal	0.4–2.8^e^	Kerenga et al. 2019
NTC	Hevein-like peptides	Antifungal	90–1250^f^	Van Parijs et al. 1991
Pn-AMP1	Hevein-like peptides	Antifungal	3–26^f^	Koo et al. 1998
Pn-AMP2	Hevein-like peptides	Antifungal	75^f^	Koo et al. 1998
pnAMP-h2	Hevein-like peptides	Antifungal	23–52^f^	Koo et al. 2002
Ep-AMP1	Knottin-type peptides	Antibacterial, Antifungal	0.31–10^e^	Aboye et al, 2015
Psacotheasin	Knottin-type peptides	Antibacterial, Antifungal	12.5–25^c^, 1.56–3.13^c^	Hwang et al. 2010a, b
bevuTI-I	Knottin-type peptides	Trypsin inhibitory Prolyl oligopeptidase	471^d^ 11^e^	Retzl et al. 2020
Tk-AMP-X1	α-hairpinin	Antifungal	7.5–30^f^	Utkina et al. 2013
Tk-AMP-X2	α-hairpinin	Antifungal	7.5–30^f^	Utkina et al. 2013
MBP-1	α-hairpinin	Antibacterial, Antifungal	30^b^	Duvick et al. 1992
Ps-LTP1	Lipid Transfer Protein	Antibacterial, Antifungal	40^e^, 10-40^e^	Bogdanov et al. 2016
McLTP1	Lipid Transfer Protein	Antibacterial	12.5-200^e^	Souza et al. 2018
Lc-LTP1	Lipid Transfer Protein	Antibacterial, Antifungal	40^e^, 5-40^e^	Bogdanov et al. 2015
Lc-LTP2	Lipid Transfer Protein	Antibacterial, Antifungal	20-40^e^, 10-40^e^	Bogdanov et al. 2015
Lc-LTP3	Lipid Transfer Protein	Antibacterial, Antifungal	40^e^, 10-40^e^	Bogdanov et al. 2015
Snakin-1	Snakins	Antibacterial, Antifungal	3^a^, 1–10^a^	Segura et al. 1999
Snakin-2	Snakins	Antibacterial, Antifungal	1-20^a^	Tavares et al. 2008
cyO2	Cyclotide family	Antibacterial, Anticancer	2-9^c^, 0.1–0.3^e^	Pränting et al. 2010; Lindholm et al. 2002
varv A	Cyclotide family	Anticancer	2.7–6.35^e^	Lindholm et al. 2002
varv F	Cyclotide family	Anticancer	2.6–7.4^e^	Lindholm et al. 2002
kalata B1	Cyclotide family	Insecticidal	6.5–21^f^	Colgrave et al. 2008
kalata B2	Cyclotide family	Insecticidal	4.7–17^f^	Colgrave et al. 2008
kalata B6	Cyclotide family	Insecticidal	2.6–7.9^f^	Colgrave et al. 2008
kalata B6	Cyclotide family	Insecticidal	17–19^f^	Colgrave et al. 2008
Poca A	Cyclotide family	Anticancer	1.8^e^	Pinto et al. 2018
Poca B	Cyclotide family	Anticancer	2.7^e^	Pinto et al. 2018
CyO4	Cyclotide family	Anticancer	9.8^e^	Pinto et al. 2018

^a^EC_50_(Effective concentration for half-maximum inhibition in µM)

^b^MIC (Minimum inhibitory concentration in µg/ml)

^c^MIC (Minimum inhibitory concentration in µM)

^d^IC_50_(half-maximum inhibitory concentration in nM)

^e^IC_50_(half-maximum inhibitory concentration in μM)

^f^IC_50_(half-maximum inhibitory concentration in µg/ml)

### Thionins

**Fig. 2:**
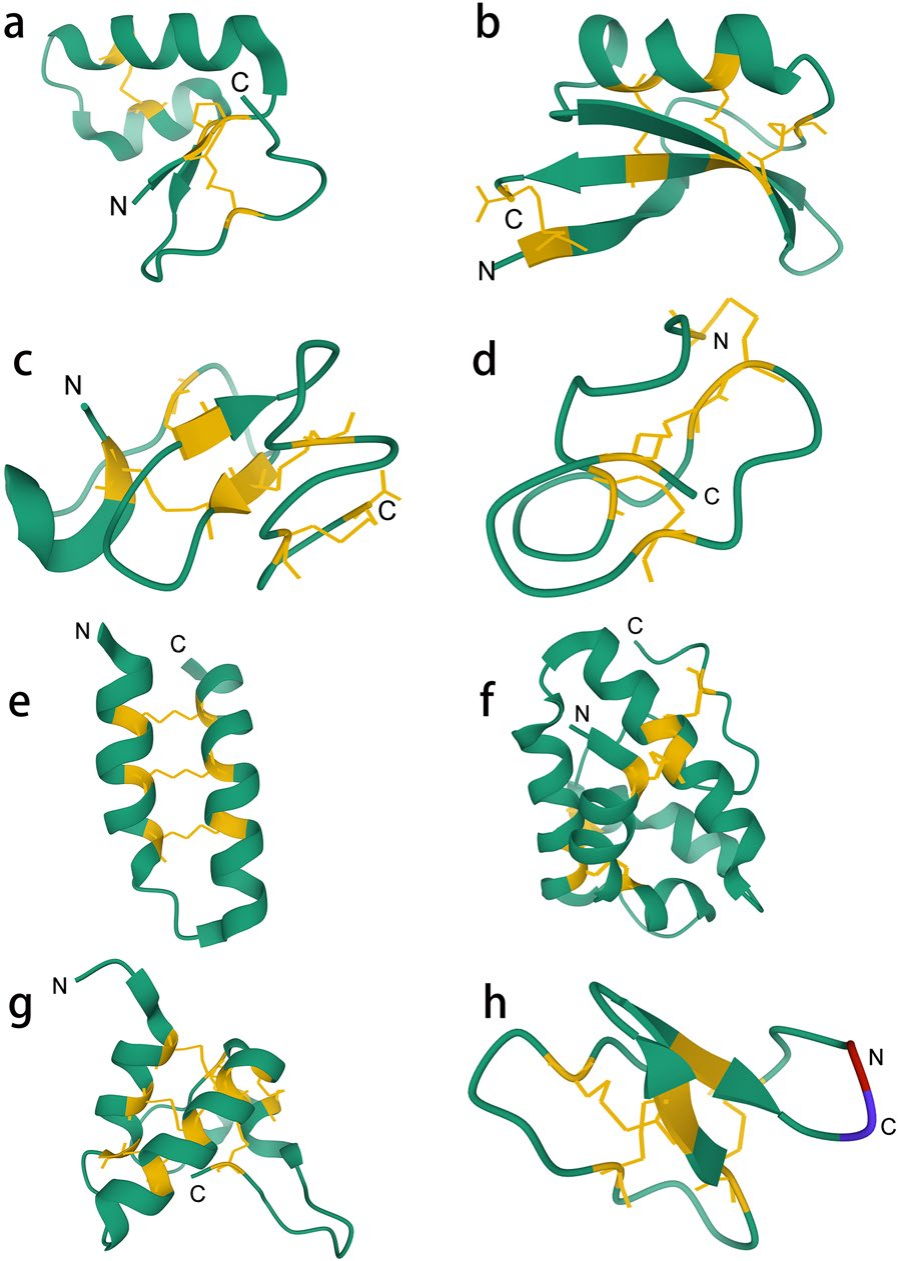
3D structures of plant AMPs. **a** thionin from *Crambe hispanica* subsp*. Abyssinica* (PDB:3U7T). **b** plant defensin from *Aesculus hippocastanum* (PDB:1BK8). **c** hevein-like peptide from *Gypsophila vaccaria* (PDB:5XDI). **d** knottin-type peptide from *Hibiscus sabdariffa* (PDB:5GSF). **e** α-hairpinin family peptide from *Nigella sativa* (PDB:2NB2). **f** lipid transfer protein from *Hordeum vulgare* (PDB:3GSH). **g** snakin from *Solanum tuberosum* (PDB:5E5Y). **h** cyclotide from *Clitoria ternatea* (PDB:2LAM). The N-terminus and C-terminus of the cyclotide are connected. Red represents the N-terminal amino acid and blue represents the C-terminal amino acid. Green represents the amino-acid skeleton. Yellow represents cysteines and disulfide bridges between cysteines

Thionins were first found in the organic solvent-extractable fraction of wheat and barley seeds as a group of cysteine-containing, amphipathic plant proteins with small molecular masses (Hughes et al. 2000); they are composed of 45–48 amino acid residues (~ 5 kDa), contain 6 or 8 cysteines, and 3 or 4 disulfide bonds (Fig. 2a) (Stec 2006). Thionins have been identified in many plant species (Hughes et al. 2000). They form a ring structure topology because of an end-to-end disulfide bond that connects the N- and C- termini; thus, they could be categorized as cyclic peptides (Tam et al. 2015). However, they are not true cyclic peptides because the disulfide-bonded cysteines are not located immediately at the N- and C-termini. Other cysteines in the polypeptides can also form disulfide bonds (Milbradt et al. 2003; Rao et al. 1995a, b). Initially, thionins were called phytotoxins because of their effects on bacteria, fungi, animal and plant cells, and insect larvae (Ebrahim-Nesbat et al. 1989; Evans et al. 1989; Fernandez et al. 1972; Schmidt et al. 2019; Taveira et al. 2014).

### Plant defensins

Plant defensins are a large antimicrobial peptide superfamily that widely exists in the plant kingdom. According to data mining of plant genomes, such as the *Arabidopsis thaliana* genome, plant defensins are the best-known and probably the largest family of all membrane-soluble plant AMPs (Graham et al. 2008). The first identified plant defensin, isolated from wheat and barley, was found to have a molecular weight of 5 kDa and contains four disulfide bonds (Fig. 2b) (Bruix et al. 1993). Similar characteristics are present in α-thionins, β-thionins, and γ-thionins. However, γ-thionins were later deemed to be structurally different from α-/β-thionins and were classified as plant defensins according to their similarities in sequence, structure, and function to mammalian and insect defensins (Broekaert et al. 1995; Mendez et al. 1990; Pelegrini and Franco, 2005). As an important class of AMPs, defensins have highly conserved structural scaffolds and can be found in almost all plants (Taylor et al. 2008). Despite their highly conserved structures, their amino acid sequences are highly variable, except for the cysteine residues that form stable disulfide bonds and some other conserved residues (Parisi et al. 2019). Generally, their three-dimensional structure consists of three antiparallel β-sheets and an α-helix parallel to the β-sheets. Plant defensins are stabilized by four disulfide bonds, including a cysteine-stable Csαβ motif (García-Olmedo et al. 1998). They possess a variety of biological functions, such as inhibiting microbial growth, inhibiting α-amylase and trypsin activity, affecting self-incompatibility, mediating abiotic stress, acting as epigenetic factors, and changing the redox state of ascorbic acid (Carvalho and Gomes 2011; Fujimura et al. 2003; Gao et al. 2000; Sitaram, 2006; Terras et al. 1995; Wijaya et al. 2000).

### Hevein-like peptides

Hevein-like peptides are alkaline peptides first identified in the latex of the rubber tree *Hevea brasiliensis* as the protein component with the highest content, and they show strong antifungal activity in vitro (Archer et al. 1960; Van Parijs et al. 1991). Hevein-like peptides contain a conservative chitin-binding domain with the amino acid sequence SXFGY/SXYGY, where X can be any amino acid residue (Beintema, 1994; Jiménez-Barbero et al. 2006; Kini et al. 2015). The hevein domain consists of an antiparallel β-sheet and a short α-helix, and the scaffold is stabilized by 3–5 disulfide bonds (Fig. 2c) (Porto et al. 2012). Similar to hevein, hevein-like peptides can inhibit the growth of chitin-containing fungi and protect plants from attack by a wide range of fungal pathogens (Beintema 1994; Van Parijs et al. 1991).

### Knottin-type peptides

Plant knottins, which were first discovered 20 years ago and contain approximately 30 amino acids, are a superfamily that includes inhibitors of the α-amylase, carboxypeptidase, and trypsin families, as well as cyclic peptides (Le Nguyen et al. 1990). Generally, knottin-type peptides are the smallest in size among plant AMPs. They can bind to a variety of molecular targets and have multiple biological functions, such as promoting resistance to biotic and abiotic stresses, stimulating root growth, acting as signaling molecules, and enhancing symbiotic interactions. They also possess antimicrobial activity against bacteria, fungi, and viruses and even show cytotoxic, insecticidal, and anti-HIV activities (Aboye et al. 2015; Hwang et al. 2010a, b; Pallaghy et al. 1994). The typical structure of knottins involves conserved disulfide bonds between multiple cysteine pairs, forming a cystine knot (Fig. 2d). The cystine motifs of plant knottins differ within different subfamilies. In different organisms, some functionally unrelated protein families have similar knottin structures. In plants, proteins such as defensins and protease inhibitors also have cystine motifs similar to those in knottin-type peptides (Gelly et al. 2004). Although plant defensins also contain a cystine-knotting motif, they differ from knottin-like peptides in their cysteine spacing (Tam et al. 2015).

### α-Hairpinin family

The α-hairpinin family represents a class of Lys/Arg-rich plant defense peptides, which are distinguished from other AMPs by special Cys motifs to form a highly characteristic helix-loop-helix secondary structure (Slavokhotova and Rogozhin, 2020). This helix-loop-helix structure contains two antiparallel α-helices. It is stabilized by two disulfide bonds in the tertiary structure (Sousa et al. 2016) (Fig. 2e). This AMP family has a spectrum of biological activities, such as antimicrobial, trypsin-inactivating, and ribosome-inactivating activities (Slavokhotova and Rogozhin 2020; Tam et al. 2015). A 33-amino acid AMP, MBP-1, isolated from maize kernels can inhibit spore germination and mycelial elongation of several phytopathogenic fungi and bacteria (Duvick et al. 1992).

### Lipid transfer proteins

Lipid transfer proteins (LTPs) are small cationic peptides with molecular weights ranging from 7 to 10 kDa. They have eight Cys residues, but their amino acid sequences have low similarity (Yeats and Rose 2008). The three-dimensional structure of the LTP family contains a conservative pattern of eight cysteines and four disulfide bonds that stabilize a tight tertiary fold consisting of four flexible loop-linked helices with hydrophobic cavities, including lipid-binding sites. Furthermore, although the eight cysteines of LTPs are conserved, changes in the motif pairs of cysteines that form disulfide bonds are observed (Fig. 2f) (Carvalho and Gomes 2007). LTPs can bind to a variety of lipids, including fatty acids, phospholipids, prostaglandin B2, hemolytic derivatives, and acyl coenzyme A (Carvalho and Gomes 2007; Liu et al. 2015; Sels et al. 2008). LTPs are classified as LTP1s (9 kDa) or LTP2s (7 kDa) according to their molecular weight. These peptides have been shown to reversibly bind and transport hydrophobic molecules in an in vitro model (Carvalho and Gomes 2007). LTPs not only inhibit the growth of fungi and bacteria, but they also participate in plant defense systems (Blein et al. 2002; Bogdanov et al. 2015, 2016; Souza et al. 2018).

### Snakins

In the plant AMP family, the snakin class contains 12 cysteine residues, having the largest number of disulfide bonds. The first defined snakin peptide, snakin-1 was found in potato tubers (*Solanum tuberosum*), and had some sequence motifs in common with snake venoms (Segura et al. 1999). These proteins are known to be cysteine-rich (~ 19% Cys) with approximately 63 amino acid residues and six disulfide bonds, whereas other types of AMPs usually have two to four disulfide bonds (Fig. 2g) (Segura et al. 1999; Tavares et al. 2008). Snakins can be constitutively or inducibly expressed by biotic or abiotic stress in different organs, such as the roots, stem, leaves, flowers, and seeds. Silencing *Snakin-1* in potato affects cell division, primary metabolism, and cell wall composition, resulting in altered potato height, leaf size, and leaf morphology (Nahirñak et al. 2012). Snakin-1 and snakin-2 were both shown to inhibit the growth of fungi (e.g., *Magnaporthe grisea*, *Fusarium solani,* and *Botrytis cinerea*) and bacteria (e.g., *Dickeya dadantii, Ralstonia solanacearum,* and *Sinorhizobium meliloti*) (Berrocal-Lobo et al. 2002).

### Cyclotide family

Cyclotides are long-chain cyclic peptides containing 28–37 amino acids produced by plants; most plant anionic AMPs belong to the cyclotide family (Harris et al. 2009; Prabhu et al. 2014). They possess a cyclic backbone consisting of six loops, which are formed by six conserved cysteine residues arranged and crosslinked in a knotted manner (called cyclic cystine knots) that are stabilized by three disulfide bonds (Fig. 2h) (Craik 2009; Ireland et al. 2006; Pränting et al. 2010). This cysteine junction is formed when the first two disulfide bonds (Cys1-Cys4 and Cys2-Cys5) and their interconnected backbone form a ring that is penetrated by the third disulfide bond, Cys3-Cys6 (Ireland et al. 2010). The cyclic cystine knot framework also endows cyclotides with high resistance to thermal and chemical denaturation as well as proteolytic degradation, making them potential therapeutic agents (Mehta et al. 2020), including antitumor (Lindholm et al. 2002), anti-HIV (Sangphukieo et al. 2015), insecticidal (Colgrave et al. 2008; Jennings et al. 2001), and antibacterial agents (Pränting et al. 2010).

## Mechanisms of plant AMP activities

At present, it is believed that AMPs exert their antimicrobial effects through their ability to interact with microbial membranes (Quemé-Peña et al. 2019; Vestergaard et al. 2017). The exact mechanism by which AMPs exert their bactericidal activity has not been fully elucidated. However, because bacterial membranes are rich in anionic lipids such as phosphatidylserine and cardiolipin, it is generally believed that positively charged peptides interact with negatively charged bacterial cell membranes, resulting in increased membrane permeability and rapid cell death (Chen et al. 2017). The mode of action of AMPs depends on their properties, such as their sequence, size, charge, hydrophobicity, and affinity (Bhattacharjya et al. 2009). Activity of these peptides can be promoted by hydrophilic, positively charged domains, which interact with negatively charged microbial membrane surfaces and the head groups of bilayer phospholipids, leading to cell membrane penetration. Thus, cell transmembrane potential and pH gradient are destroyed, osmotic regulation is affected, and cell respiration is inhibited, causing microbial death. Studies have shown that the ability of a peptide to bind to nonspecific regions on the membrane of a receptor or target cell depends directly on the peptide's intrinsic or dynamic conformation, with transitional steps occurring before or during binding (Dorschner et al. 2006). There are four models of interaction between an AMP and cell membrane: barrel-stave pore, carpet mechanism, toroidal pore, and disordered toroidal pore (Brogden 2005; Cirac et al. 2011; Nguyen et al. 2011).

### Barrel-stave pore

**Fig. 3:**
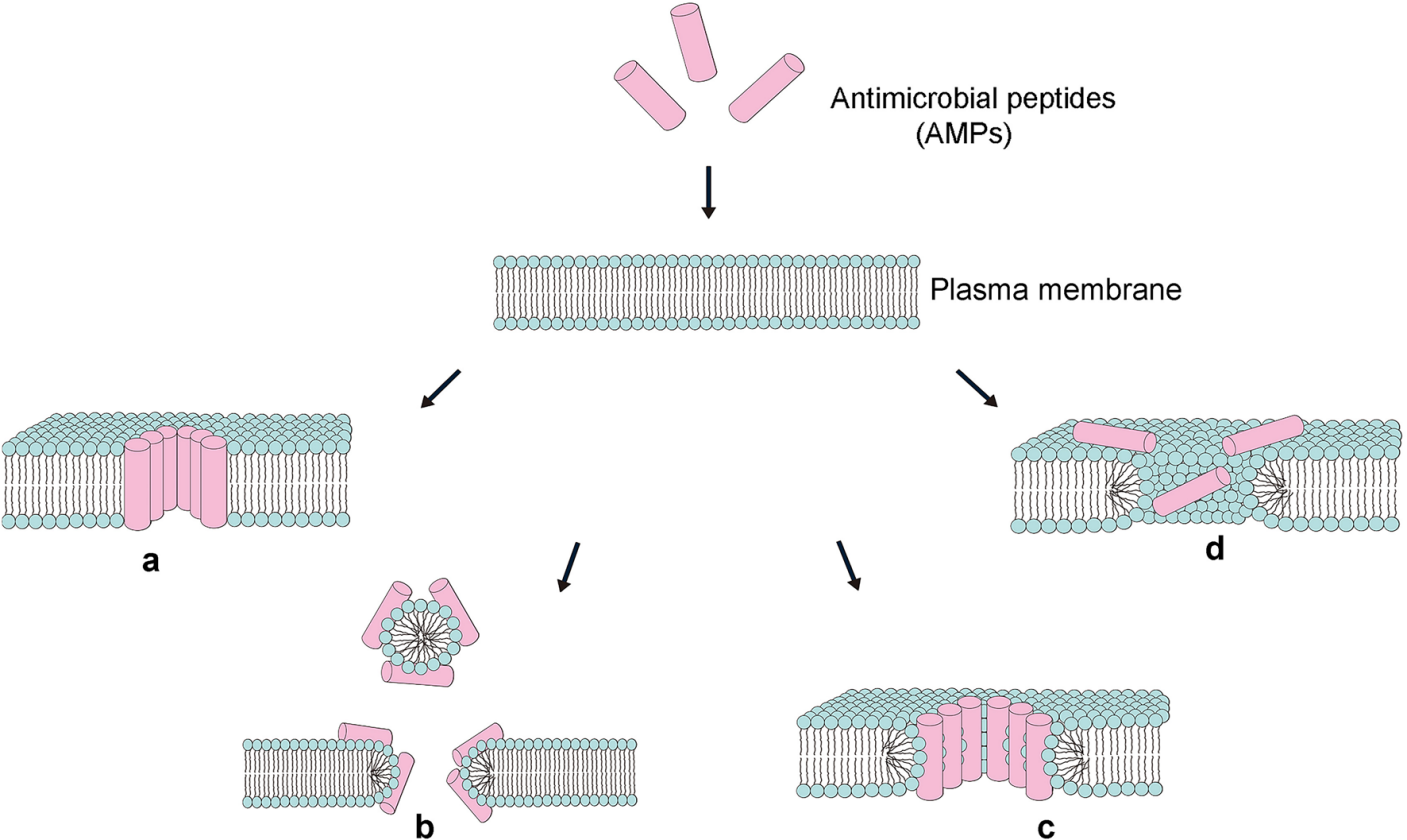
The four major mechanisms of AMP activity. When the contact between AMPs and the bacterial plasma membranes reaches a critical level, the following four interactions can occur between the AMPs and the plasma membranes. **a** barrel-stave pore: AMPs insert vertically in the plasma membrane to form transmembrane pores. **b** carpet mechanism: Peptides are adsorbed parallel to the lipid bilayer to cover the cell surface, causing membrane rupture. **c** toroidal pore: an intermediate type between the carpet mechanism and the barrel-stave pore.Peptides and lipids form the pores. **d** disordered toroidal pore: Pore formation is more random and involves fewer peptides, but additional peptides are required to stabilize the opening

According to this model, AMPs first attach to the surface of a lipid membrane at an axis parallel to its surface. As the hydrophobic region of the peptide aligns with the hydrophobic core of the lipid bilayer, a permanent transmembrane pore is formed, and the hydrophilic region of the peptide constitutes the inner part of this pore. AMPs insert vertically in the bilayer, bind, and form a pore, and these peptides are arranged in the pore cavity parallel to the phospholipid chains, remaining perpendicular to the bilayer plane (Fig. 3a). This model predicts a regular formation of AMP aggregates that interact with and maintain contact with membranes, and there is evidence that after pore formation, some AMPs enter the cell and interact with specific intracellular components (Travkova et al. 2017).

### Carpet mechanism

According to this model, the peptide is adsorbed parallel to the phospholipid bilayer to produce a detergent-like effect. After achieving sufficient coverage, the amphiphilic peptides form cyclic aggregates with the membrane lipids, leading to rupture of the membrane. Specific peptide–peptide interactions are not required. AMPs diffuse across the lipid membrane to form a parallel arrangement, resulting in the loss of directionality of the accumulated lipid molecules and their disruption into small aggregates (Fig. 3b). The carpet model leads to membrane rupture without involving internalization of AMPs (Järvå et al. 2018).

### Toroidal pore

The toroidal pore model is an intermediate type somewhere between the carpet and barrel-stave pore mechanisms. Once the AMP molecule is adsorbed to the bilayer surface and structurally transformed, the membrane bends, and then, the peptide passes through the bilayer to form a pore (Fig. 3c). Peptide molecules form the inner layer of the core and remain in contact with the lipid heads and the water core (Wimley 2010).

### Disordered toroidal pore

For this model, after the formation of a random pore on the plasma membrane, the lipids twist inward, and the pore cavities are arranged by phospholipid head groups. The deeply embedded peptides stabilize straight circular pores, whereas the remaining peptides are arranged at the openings of the pores and stabilize the curvature of the membrane (Fig. 3d) (Cirac et al. 2011).

It is generally believed that the electrostatic attraction between cationic AMPs and the negatively charged microbial cell surface is an important factor in their antimicrobial activity, which would appear to exclude anionic peptides. However, it has been found that, similar to cationic antimicrobial peptides, anionic antimicrobial peptides adopt amphiphilic α-helixes and β-sheets and can interact with microbial membranes (Parisi et al. 2019). The anionic antimicrobial peptide AP2 has a net negative charge but AP2 has some regions containing cationic amino acid residues on its amphoteric α-helix. These regions could considerably promote the interaction between anionic antimicrobial peptides and negatively charged structures exposed on the surface of bacterial membranes (Laverty et al. 2011; Sowa-Jasiłek et al. 2020).

Membrane targeting of AMPs was initially believed to be the only mode of action, but there is now increasing evidence that AMPs can act as antimicrobial agents without interacting with the membrane (Ulm et al. 2012). As many microorganisms can survive even after a large area of the membrane has been damaged, studies from this perspective have found that AMPs can also target key processes leading to bacterial death. Examples include inhibition of cell wall components, DNA or protein synthesis, protein folding and metabolic turnover, without damaging the cell membrane (Aoki and Ueda 2013). Simultaneously, a non-soluble mechanism could be used to kill bacteria. In this non-soluble mechanism, AMPs penetrate the adventitia and utilize protein-mediated transmembrane transport. AMPs can also inhibit protein synthesis by targeting ribosomes (Sharma et al. 2016). This further indicates that AMPs can induce cell death without involving membrane-breakdown mechanisms (Rehal et al. 2019).

## Main parameters influencing the antimicrobial activities of plant AMPs

The structure–activity relationship analysis of plant AMPs indicated that their amino acid residues, net charge, hydrophobicity, amphipathicity and structural features are the most important physicochemical and structural parameters for their antimicrobial activity (Bhattacharjya et al. 2009). In addition to these main factors, some external factors, such as pH, temperature, and metal ions, also affect the activities of plant AMPs. It is worth noting that all of these factors are interrelated, and a change in one factor would lead to concomitant but inadvertent alterations in others.

### Amino acid residues

In general, AMPs are classified on the basis of their net charge as cationic peptides rich in arginine or lysine, and anionic AMPs rich in aspartic acid or glutamic acid. The amino acid sequence has a characteristic influence on the structure and function of the peptide. Changes in amino acid sequence, length, and net charge will affect the hydrophobicity of the short amphiphilic peptide and directly affect its antibacterial activity and cytotoxicity (Gong et al. 2019; Sprules et al. 2004). Some AMPs with multiple Arg residues may be internalized via the anionic sulfated glycosaminoglycan pathway, and Arg-lacking AMPs were reported to not interact with sulfated glycosaminoglycans (Poon et al. 2007; Tang et al. 2013; Torcato et al. 2013). Arginine can provide positive charges and forms a large number of electrostatic interactions compared to lysine. A previous study showed that variations in the levels of four amino acid residues, leucine, alanine, glycine, and lysine, in different host defense peptide families modulate peptide activities (Wang 2020). Introducing proline into some AMPs and the location of the proline are determining factors of AMP antitumor and antimicrobial activities as well as other bioactivities (Yan et al. 2018). Aspartic acid and glutamic acid residues in the anionic peptides can facilitate the binding of metal ions, which is necessary for their antimicrobial activity (Dashper et al. 2005). In addition, aromatic residues (mainly tryptophan) may be important determinants to anchor the antimicrobial peptides onto membranes (Fimland et al. 2002).

### Net charge

It is known that most antimicrobial peptides possess a net positive charge, and this positive charge is believed to play a major role in the interaction between the antimicrobial peptides and negatively charged membrane phospholipids. This relationship between biological activity and charge is not linear, and there are some examples of direct, indirect, or even inverse relationships between the charge and biological activity. An increase in the charge of AMPs will increase their antibacterial activity against gram-negative and gram-positive pathogens, but a threshold was found beyond which an increase in the positive charge no longer augments this activity. An excessively high net charge will lead to increased hemolytic propensity and decreased antimicrobial activity (Dathe et al. 2001; Jiang et al. 2009; Wang et al. 2019).

### Hydrophobicity

Hydrophobicity is another necessary parameter to ensure the antibacterial efficacy and cell selectivity of AMPs. However, some studies have shown that the hemolytic activity of AMPs increases with enhanced hydrophobicity (Liscano et al. 2019). The higher hydrophobicity of AMPs could increase their ability to penetrate deeper into the hydrophobic core of cell membranes. Studies have shown that increasing AMP hydrophobicity is generally associated with increasing antimicrobial activity within a certain range. Increasing the hydrophobicity of the hydrophobic face will increase the antimicrobial activity of AMPs. When the peptide-length-dependent threshold is exceeded, the hemolytic activity of an AMP significantly increases and its cell selectivity decreases (Gong et al. 2019; Uggerhøj et al. 2015).

### Alpha-helix and amphipathicity

The α-helix is the most common conformation of the various secondary structures in AMPs. Amino acid substitutions that significantly damage the helical structures in peptides can lead to a decrease in antimicrobial activity (Lee et al. 2016). Most helical AMPs that adopt the oblique-orientated α-helical configuration invade microbial membranes partially, resulting in membrane destabilization and promoting effects such as membrane fusion, hemolysis, and the formation of non-bilayer lipid structures (Dennison et al. 2005; Gong et al. 2019; Juretić et al. 2019). The amphipathic nature of AMPs is closely related to the formation of α-helical structures. The helix spatially segregates hydrophilic and hydrophobic residues on opposing faces along its long axis, leading to the formation of amphiphilic structures. When AMPs interact with the bacterial membrane, the ability to maintain a balance between amphiphilic and hydrophobic properties is also responsible for the biological activity of oblique-orientated α-helices (Harris et al. 2006; Liang et al. 2020). Optimizing amphiphilicity without changing other structural parameters resulted in significantly increased bactericidal activity and cytotoxicity due to strengthened hydrophobic interactions and membrane affinity (Takahashi et al. 2010).

### Other factors

In addition to the major factors mentioned in previous sections, there are many minor factors that need to be mentioned. One study has shown that the dimerization of β-sheet peptides could also increase the antimicrobial activity of AMPs by promoting a deeper penetration into the hydrophobic membrane core than would be allowed by monomeric peptides (Teixeira et al. 2012). The addition of metal ions can cause conformational helix changes, which can affect the hydrophobic region of the helix and AMP activity (Oard et al. 2006). Although anionic peptides are composed entirely of negatively charged residues, some AMPs can interact with microbial membranes by co-opting cationic metal ions to form salt bridges (Dashper et al. 2005; Dennison et al. 2018). pH plays a variable role in the interaction of AMPs and microbial membranes. Some studies have shown that a change in pH can significantly affect the antibacterial activity of AMPs, but pH can also affect the membrane lipid composition of bacteria and increase their resistance to AMPs (Dennison et al. 2016; Koo et al. 1998). It has been found that disulfide bonds and hydrogen bonds contribute to the stability of native-folded AMPs, and both types of bonds affect the activity of AMPs by influencing their folding stability (Ranade et al. 2020; Vila-Perelló et al. 2005). In addition to the chemical bonds mentioned in previous sections, a few others have been reported, such as thioether bonds, which are required for peptide maturation (Pham et al. 2020; Wieckowski et al. 2015). However, the structure–activity relationship between these chemical bonds and AMPs is not clear. Future studies are required to more closely examine this relationship.

## Application potential of plant AMPs

### Plant AMPs and human diseases

The pharmaceutical industry represents the most important application area for AMPs. Antibiotics, which can cure many infectious and fatal diseases, have always been crucial for human health in the treatment of pathogenic bacterial infections. Over the years, many pathogenic bacterial strains have evolved and become resistant to existing antibiotics, which is a major problem and challenge faced by microbiologists and pharmacologists engaged in antibiotic production (French 2005). One of the main reasons for the prevalence of antibiotic resistance is the widespread use of antibiotics in humans and animals; overuse leads to spontaneous mutations in antibiotic targets and the exchange of plasmids encoding resistance genes. Therefore, in the severe situation of ongoing resistance of pathogenic bacteria to antibiotics, medical institutions around the world are urgently looking for alternatives to general antibiotics (Breithaupt 1999). Accordingly, people have turned their attention to AMPs. Because most AMPs can target pathogens in a nonspecific way and the direct interaction of AMPs with the biofilm of pathogenic bacteria increases biofilm permeability to antibiotics, AMPs can be combined with traditional antibiotics to produce a synergistic effect. Meanwhile, the significant advantage of AMPs lies in their universal mechanism of action, which is significantly different from that of traditional antibiotics (Cassone and Otvos 2010). AMPs have broad application prospects as promising alternatives to antibiotics for the treat of human diseases-resistant infections. The bacterial surface is negatively charged due to lipopolysaccharides and teichoic acids, while the mammalian cell surface contains amphoteric phospholipids, cholesterol, and sphingomyelin. Generally, positively charged AMPs interact easily with negatively charged bacterial biofilms (Bhattacharjya et al. 2009; Hilchie et al. 2013). This suggests that AMPs can specifically target cell membrane of pathogen, which may reduce the negative effects of AMPs on the human cell. Meanwhile, the study has shown that the Ep-AMP1 from *Echinopsis pachanoi* has low human-cell cytotoxicity, relative to the human antimicrobial peptide LL-37 (Aboye et al. 2015; Kerenga et al. 2019).

Studies have confirmed that AMPs in the human body act as multifunctional effectors of the innate immune system. In addition to their direct antimicrobial function, they have immune regulatory functions (Ganz 2003). Increasing evidence suggests that AMPs can influence human immune responses to a variety of diseases by influencing signaling in the human body, which also indicates the potential of AMPs in the treatment of a variety of high-fatality diseases. For example, AMPs can simultaneously regulate multiple signaling pathways, including inhibition of the synthesis of the signaling molecules, reactive oxygen species (ROS) and nitric oxide (NO), modification of mitogen-activated protein kinase (MAPK) signaling, and alterations of wound and vascular healing (Choi et al. 2012). At present, studies have also confirmed that peptides extracted from chickpea can significantly inhibit the activities of fatty acid synthase (FAS) and 3-hydroxy-3-methylglutaryl coenzyme A reductase (HMGR). Chickpea peptides can significantly reduce serum total cholesterol (TC), triglyceride (TG), and low-density lipoprotein cholesterol (LDL-C) contents and increase serum high-density lipoprotein cholesterol (HDL-C) content in obese rats induced by high-fat diet, and the peptides significantly reversed the blood and liver metabolic disorders in these obese rats (Shi et al. 2019). Poca A and poca B are cyclotide AMPs extracted from the root tissues of *Pombalia calceolaria*; they have been found to exhibit a strong inhibitory effect on MDA-MB-231 breast cancer cells (Pinto et al. 2018). In addition, peptide fractions of agglutinin and abrin from *Abrus* spp. show potential for tumor treatment and immune stimulation. However, due to the apoptotic activity of their components on mammalian cells, *Abrus* spp. are highly toxic plants. This indicates that both medicinal plants and even plants that are toxic and harmful to human beings have the potential to produce AMPs (Bhutia et al. 2019; Mukhopadhyay et al. 2014).

### Application of AMPs in agricultural production

In agriculture, in order to cope with plant diseases and insect pests and to improve crop yields, several chemical pesticides have been applied during crop production. With the continuous use of these chemical pesticides, the harm of chemical residues to humans and animals is becoming increasingly obvious. In this case, the application of AMPs as natural, low-toxicity, and high-efficiency antimicrobial proteins in plants has received increased attention. Most natural plant AMPs are encoded by specific genes that are constitutively expressed at basal levels and are rapidly transcribed after being induced by pathogens. In response to pathogen stimulation, multiple AMPs can be found simultaneously in different organs of the same plant (Mith et al. 2015). Most AMPs can target pathogens in a nonspecific way, and pathogens do not easily develop resistance to AMPs, which makes AMPs very suitable for developing plant disease resistance; the ability of AMPs to fight pathogens has been demonstrated by the expression of heterologous plant AMPs in transgenic plants (Goyal and Mattoo 2014). Many transgenic plants expressing heterologous AMPs are protected from pathogens. For example, the expression of *Mirabilis jalapa* defensin Mj-AMP1 in tomato enhanced tomato resistance to *Alternaria solani* (Schaefer et al. 2005), the expression of radish defensin Rs-AFP2 in tobacco and tomato helped the plants resist *Amanita longipes* infection (Terras et al. 1995), and hevein Pn-AMP from *Pharbitis nil* protected tobacco against *Phytophthora parasitica* (Koo et al. 1998, 2002). Therefore, AMPs with low toxicity and high efficiency relative to chemical fungicides have become a good choice for plant disease control.

Through many in-depth studies of the functions of AMPs in plants, cumulative evidence has suggested that AMPs play other roles in addition to acting as antimicrobial proteins in plant innate immunity. AMPs seem to be involved in all stages of the plant life cycle, from seed germination to root development, and from the growth and development of vegetative and reproductive organs to the promotion of reproduction, seed development, and seed longevity (Stotz et al. 2009; Marshall et al. 2011). Studies on silencing or overexpression of the defensin DEF2 in tomatoes showed that it could significantly affect tomato pollen viability, seed production, and plant morphology (Stotz et al. 2009). ZmES1, a cysteine-rich defensin-like protein in maize, is able to interact with the potassium channel KZM1, leading to the rupture of maize pollen tubes to expel sperm (Amien et al. 2010). In radish, the defensins RsAFP1 and RsAFP2 are released simultaneously when the seed coat opens to promote germination (Terras et al. 1995). Accumulation of VvAMP1 defensin transcripts in grape tissues during fruit ripening showed tissue and developmental stage specificity (de Beer and Vivier 2008). Phytohormones are key regulators of plant growth and development and resistance to pests and diseases. They are essential signaling molecules for plants to sense changes in the external environment, self-regulate their growth, resist adverse environments, and survive. The production and accumulation of AMPs in plants are also regulated by various phytohormones. Thi2.1 is an abundant thionin in *Arabidopsis* pollen and horn fruit, which can be induced by pathogens (e.g., *Fusarium oxysporum*), wounds, and the plant defense hormone jasmonic acid (JA) (Báez-Magaña et al. 2018). JA has also been shown to regulate expression of the hevein-like AMP, WJAMP-1, in sunflower leaves (Kiba et al. 2003). The tomato defensin, tgas118, appears to be regulated by gibberellin throughout flower development (van den Heuvel et al. 2001). In addition to plant growth and development status and biological stress, phytohormone expression can be regulated by many abiotic stresses, such as drought, cold, and saline–alkaline stress, which also suggests that plant AMP expression can be affected by such abiotic stresses (Lay and Anderson 2005). There is a class of AMPs in *Triticum kiharae*, the α-hairpinin TK-AMP, which can be induced by abiotic and biological stresses (Utkina et al. 2013). These studies show that AMPs not only act as innate immune factors against pathogenic bacteria in plants, but also have the ability to enhance plant resistance to pests, diseases, and stress and to promote better growth and development of plants in agricultural production.

### Application of AMPs in food and breeding industries

The abuse of antibiotics has made bacterial infections a major concern in developed countries. Besides the use of antibiotics in the treatment of human diseases, antibiotics have been widely used in the food and breeding industries. However, when antibiotics are utilized in the food industry and aquaculture, their residues may impact the taste of foods and may cause harm to human beings. The presence of residues in feeds can lead to antibiotic resistance in the microorganisms of livestock and the evolution of resistance in zoonotic bacteria, leading to concerns that drug resistance may shift from livestock to humans (Ben et al. 2019). Both concerns have caused consumers to worry about food safety-related issues. Therefore, the development of safe and effective antibiotic substitutes is equally important for the food and breeding industries. AMPs from food proteins have potential as food additives because they have little influence on the physical and chemical properties of foods and they exert antimicrobial activity at low concentrations (Ahmed and Hammami 2019). Studies have confirmed that the engineered modification of food-grade *Lactococcus lactis* can produce AMPs with inhibitory effects on a variety of pathogenic microorganisms such as *Staphylococcus aureus*, *Enterococcus faecalis*, *Listeria monocytogenes*, *Pseudomonas aeruginosa*, *Escherichia coli*, and *Salmonella typhimurium*. *L. lactis*, as a recognized food safety bacterium, can produce heterologous and active AMPs, which makes it a potential food preservative in dairy products and starter cultures during fermentation (Tanhaeian et al. 2020). AMPs can be used in the breeding industry to improve the growth performance of cultured animals, promote the digestion of animal nutrients and intestinal health, change the intestinal flora, and enhance immune function, among other applications. It is generally believed that the beneficial effects of AMPs on growth performance are mainly due to their antimicrobial and immunomodulatory activities, thus promoting the digestion and health contribution of nutrients. Other advantages include low toxicity, low residues, and the difficulty of pathogenic bacteria developing resistance to them in cultured animals (Hu et al. 2013; Wang et al. 2016). Therefore, AMPs can also be used in the food and breeding industries as safe and effective alternatives to antibiotics. Although the current applications of AMPs mainly focus on the treatment of human diseases and improvement of agricultural production, we should not ignore their applications in the food and breeding industries. As one of the main sources of natural AMPs, the use of plant AMPs in these industries is still a subject of great research interest.

## Conclusion and expectations

AMPs are a class of small molecule proteins with broad-spectrum antimicrobial properties. As an integral part of plant innate immunity, the advantages of plant AMPs are their broad-spectrum antimicrobial activity and low toxicity to eukaryotic cells. AMPs produced by plants are diverse, and the classification of AMPs in plants is mainly based on their sequences and structures. They are mainly divided into eight types: thionins, plant defensins, hevein-like peptides, knottin-type peptides, α-hairpinins, lipid transfer proteins, snakins, and cyclotides (Table 1). The antimicrobial activity of AMPs is their main function. The main mechanism of this antimicrobial action is believed to be the interaction between the cationic AMPs and the anion-rich plasma membranes of pathogenic bacteria. The models of AMP interaction with the membrane mainly include the barrel-stave pore, carpet mechanism, toroidal pore, and disordered toroidal pore. However, accumulating evidence shows that in addition to acting on the plasma membrane, AMPs can directly target intracellular sites to affect the normal physiological activities of pathogenic bacteria, thereby achieving antimicrobial effects. In addition to their antimicrobial functions, plant AMPs can participate in the regulation of plant growth and development, and some AMPs have shown excellent therapeutic effects on certain human diseases. Many factors can affect the antimicrobial activity and cell selectivity of AMPs, such as their amino acid residues, net charge, hydrophobicity, amphipathicity, and structural propensity. Plant AMPs also have potential as excellent alternatives to traditional antibiotics in the food and breeding industries. Thus, plant AMPs are promising candidates in agricultural production, the food industry, breeding industry, and medical fields.

There are still many problems that need to be overcome to utilize plant AMPs fully. First, AMPs can be produced in almost all plant organs. The diversity of plants and organs, as well as the diversity of screening, identification, and purification methods make the purification of plant AMPs very complex and time-consuming (Tang et al. 2018). Second, the plant AMPs produced by cultivated grains tend to have lower antimicrobial activities than those produced by wild grains. This was reported to occur because of the higher variability in C-terminal fragment sequences and higher percentage of hydrophobic amino acids in the AMPs from wild grains than those from cultivated grains, which makes it very difficult to produce active plant AMPs in large quantities (Rogozhin et al. 2018). Finally, because different plants have different cultivation conditions, sometimes we may need to express an AMP from a plant that is difficult to cultivate artificially on a large scale using transgenic technology. The AMP degradation activity of proteases present in leaf intercellular fluid may be the key to achieving expected transgene function under these conditions. Although studies have demonstrated that AMP modification by single amino acid substitution can reduce the endogenous degradation of AMPs by the proteases in leaf cells, whether such single amino acid substitutions affect AMP function remains unknown (Owens and Heutte 1997). In summary, the functional characteristics and application methods of plant AMPs still need to be studied in depth.
